# Editorial: Behavioral outcomes of traumatic brain injury

**DOI:** 10.3389/fnbeh.2022.1010395

**Published:** 2022-09-07

**Authors:** Christopher M. Olsen, Amy A. Herrold, Alana C. Conti, Cole Vonder Haar

**Affiliations:** ^1^Department of Pharmacology and Toxicology, Neuroscience Research Center, Medical College of Wisconsin, Milwaukee, WI, United States; ^2^Department of Neurosurgery, Medical College of Wisconsin, Milwaukee, WI, United States; ^3^Research Service, Edward Hines Jr., VA Hospital, Hines, IL, United States; ^4^Department of Psychiatry and Behavioral Sciences, Northwestern University, Feinberg School of Medicine, Chicago, IL, United States; ^5^John D. Dingell VA Medical Center Detroit, MI, United States; ^6^Department of Psychiatry and Behavioral Neurosciences, Wayne State University School of Medicine, Detroit, MI, United States; ^7^Department of Neuroscience, Ohio State University, Wexner Medical Center, Columbus, OH, United States

**Keywords:** traumatic brain injury, behavior, blast TBI, lateral fluid percussion injury, closed head injury, rotational injury

Traumatic brain injury (TBI) is a leading cause of death and disability for all age groups in the United States and across the globe (Johnson and Griswold, 2017; Maas et al., 2017). TBI patients often experience significant and persistent impairments in cognitive and emotional function that decrease quality of life. This special issue addresses these TBI-associated behavioral changes with a series of manuscripts that leverage rodent models of brain injury and clinical work to improve translation of preclinical studies and rehabilitation of TBI patients.

Several studies in this issue report behavioral impairments following repeated TBI. Baskin et al. found that three blast injuries (24 h apart) increased motivation to obtain sucrose pellets in male mice, which was also associated with increased compulsive or perseverative responding and impaired cognitive flexibility 1–2 months post-injury. Repeated blast injury also led to aberrant emotional behavior in rats, as reported by Dickerson et al. When male rats sustained three blast injuries that were separated by 1 h each, anxiety-like behavior was elevated 2- and 52-weeks post-injury. Deficits in social behavior also occurred at 36 weeks post-injury. These behavioral deficits were correlated with changes in astrocyte and neuronal pathology in the motor cortex and regions of the hippocampus. Using a repeated rotational injury model, Stemper et al. examined the cumulative effects of multiple subconcussive rotational injuries (scaled from studies of contact sport athletes) and found that circulating neurofilament light (NFL) served as a dose-dependent biomarker of injury, while only the highest exposure led to behavioral disturbances and glial pathology in the amygdala.

Several studies also identified variable or discrepant behavioral outcomes after TBI. Using a repeated closed-head injury model, Vonder Haar et al. identified robust impairments in attention, but altered impulsivity that varied depending on the measurement used. Injury was also associated with elevated microglial activation in the frontal cortex and nucleus accumbens, regions that can regulate attention and behavioral inhibition (Cardinal et al., 2002; Winstanley et al., 2006; Pattij et al., 2007). Fitzgerald et al. used a combination of Pavlovian associative learning, Barnes maze, and fear conditioning to assess memory recall in male and female mice 2–4 months after lateral fluid percussion injury. They identified deficits in anterograde memory that did not have retrograde memory deficits, an effect specific to male mice. Tucker and McCabe composed a review of the use of behavioral testing of anxiety-like behaviors in preclinical TBI studies, highlighting the lack of consistent results obtained, even within the same injury models. They offered guidance for improving the measurement of anxiety-like behavior in the study of TBI, including considerations for the ability of animals to properly perform the test, use of testing batteries, and reporting of common data elements. They further encouraged the use of statistical methods that are sensitive to individual differences, which are often observed in clinical and preclinical TBI studies (Lim et al., 2015; Scholten et al., 2016; Popovitz et al., 2021).

In a similar manner to Tucker and McCabe, but with focus on human patients, Duff et al. presented challenges and opportunities for improving the completeness of TBI patient registries, with the appreciation that multidimensional and longitudinal analyses could improve our understanding of individual differences in TBI outcomes in efforts to improve treatment and clinical practice (Lu et al., 2012; Nelson et al., 2018; Covington and Duff, 2021). This special issue also includes a clinical trial treatment study by Pennington et al. who tested combined exercise and virtual reality based cognitive training on cognitive function and alcohol-associated factors. Exercise, gaming, and exercise combined with virtual reality cognitive training each improved distinct aspects of cognition, while exercise alone additionally reduced alcohol craving and intake.

This special issue represents a translationally-focused collection of papers centered on the role of behavior after TBI. The reviews comment on the state of the science and future directions in functional outcomes for both preclinical and clinical TBI. Several preclinical reports implemented a range of behavioral assays to better understand the multitude of behavioral symptoms associated with TBI and better understand their underlying mechanisms. Finally, one clinical study highlighted a promising treatment for executive dysfunction among those with co-occurring alcohol use disorder and TBI. This collection underscores the range of cognitive and emotional deficits related to TBI and advances in our scientific understanding as well as areas with opportunities to develop better treatments that support TBI recovery.

## Author contributions

All authors listed have made a substantial, direct, and intellectual contribution to the work and approved it for publication.

## Conflict of interest

The authors declare that the research was conducted in the absence of any commercial or financial relationships that could be construed as a potential conflict of interest.

## Publisher's note

All claims expressed in this article are solely those of the authors and do not necessarily represent those of their affiliated organizations, or those of the publisher, the editors and the reviewers. Any product that may be evaluated in this article, or claim that may be made by its manufacturer, is not guaranteed or endorsed by the publisher.
